# What influences aromatase inhibitor continuation intention among breast cancer survivors?

**DOI:** 10.4069/kjwhn.2021.01.19

**Published:** 2021-03-18

**Authors:** Young Kyung Seo, Jeongok Park, Jin-Hee Park, Sue Kim

**Affiliations:** 1 College of Nursing, Yonsei University, Seoul, Korea; 2 College of Nursing, Mo-Im Kim Nursing Research Institute, Yonsei University, Seoul, Korea; 3 College of Nursing, Research Institute of Nursing Science, Ajou University, Suwon, Korea

**Keywords:** Aromatase inhibitor, Breast cancer, Medication adherence, Musculoskeletal pain

## Abstract

**Purpose:**

Aromatase inhibitors (AIs) are widely prescribed for postmenopausal women with breast cancer and are known to cause musculoskeletal pain. This study aimed to identify factors associated with AI continuation intention among breast cancer survivors (BCS).

**Methods:**

A cross-sectional survey was conducted on 123 BCS (stages I–III), who had been taking AIs for at least 6 weeks. Participants were recruited from a cancer center in Goyang, Korea, from September to November 2019. Descriptive statistics, Welch analysis of variance, Pearson correlation coefficients, and simple linear regression were used for the analysis.

**Results:**

Beliefs about endocrine therapy was a significant predictor of AI continuation intention (β=.66, *p*<.001). The majority of participants (87.0%) reported experiencing musculoskeletal pain since taking AIs and the score for the worst pain severity within 24 hours was 5.08±2.80 out of 10. Musculoskeletal pain, however, was not associated with AI continuation intention. Fear of cancer recurrence (FCR) was clinically significant (≥13) for 74.0% of the respondents (mean, 17.62±7.14). Musculoskeletal pain severity and pain interference were significantly associated with FCR (r=.21, *p*<.05; r=.35, *p*<.01, respectively). Pain interference was significantly associated with beliefs about endocrine therapy (r=–.18, *p*<.05).

**Conclusion:**

AI continuation intention can be modified by reinforcing patients’ beliefs about endocrine therapy. Musculoskeletal pain may have a negative effect on beliefs about endocrine therapy and increase FCR among BCS. Thus, awareness of musculoskeletal pain during AI therapy should be raised and further research is required to develop multidisciplinary pain management strategies and clinical guidelines to reinforce beliefs about endocrine therapy.

## Introduction

Aromatase inhibitors (AIs) have been used as the first choice of adjuvant endocrine therapy for postmenopausal women over the last 15 years since they are more effective than tamoxifen for postmenopausal breast cancer, and AI prescriptions account for 64% of endocrine therapy among women with invasive breast cancer [1]. After menopause, estrogen production by the ovaries terminates and estrogen is synthesized by aromatase in peripheral tissues. Hence, suppression of estrogen is more effectively achieved by using AIs, as they primarily block most of the production of estrogen.

To take full advantage of endocrine therapy, it is important to take the medication regularly throughout the recommended period [2]. However, issues with endocrine therapy adherence remain an unsolved problem. Breast cancer survivors (BCS) make a decision to continue or discontinue AI therapy every day and can reverse their decision at any time. Beryl and colleagues observed adjuvant endocrine therapy adherence among 35 BCS for 280±96 days. None of the patients started and continued medication based on a concrete decision, and only four patients were still taking the medication, without any reservations, at the final interview. Moreover, 68% of them stated that their decision could change at any time regardless of whether they were taking the medication [3]. This implies that assessing AI continuation intention is as important for maximizing AI adherence as assessing adherence itself. AI continuation intention refers to an intention to continue AI therapy and is conceptually mirrored by discontinuation intention, such as “uncertainty about persisting” [4] or “thought about stopping endocrine therapy” [5]. Since intention to take medication is a known predictor of medication adherence [6], it is expected that AI discontinuation can be prevented or altered by modifying or reinforcing intention during the decision-making process.

Newly developed or aggravated musculoskeletal pain, a major adverse effect of AI, is frequently reported as a cause of patient-driven AI discontinuation, unlike tamoxifen [7,8]. According to Lombard et al. [8], 82% of BCS taking AI as adjuvant endocrine therapy reported AI-associated musculoskeletal pain and 68% of nonadherent participants discontinued AI due to musculoskeletal pain. In Korea, a retrospective medical record study reported AI-associated arthralgia in 23% of BCS [9]. However, its relation to patient-driven discontinuation was not evaluated. Studies on AI adherence and AI-associated musculoskeletal pain have mainly been conducted in the United States, Europe, and Australia. Since musculoskeletal pain has been blamed for AI discontinuation, international research assessing the prevalence and risk factors of musculoskeletal pain has been actively conducted and is expanding to include the development of interventions to alleviate pain and improve the AI continuation rate [10]. Nevertheless, research on the self-reported incidence of musculoskeletal pain and its relation to AI continuation intention has not yet been conducted in Korea.

In addition to adverse effects, previous studies have found that endocrine therapy continuation was also associated with fear of cancer recurrence (FCR) [4] and beliefs about endocrine therapy [4,11,12]. Pain is known to amplify FCR in BCS [13] and a study conducted with breast cancer patients undergoing AI therapy showed that FCR was higher in patients who experienced musculoskeletal pain than in those who did not [14]. However, the relationship of pain to AI continuation was not evaluated. FCR plays a role in maintaining endocrine therapy among BCS with weak beliefs about endocrine therapy [4]. Beliefs about endocrine therapy has been reported to be the most powerful and important factor affecting the continuation of endocrine therapy [12] but can be negated by the adverse effects of endocrine therapy [4,11,15]. However, previous studies regarding beliefs about endocrine therapy were predominantly conducted among BCS taking tamoxifen and included only a small proportion of patients taking AIs. Thus, the roles of beliefs about endocrine therapy and FCR among patients receiving AI therapy have yet to be elucidated.

Therefore, this study aimed to identify (1) whether AI continuation intention differed according to whether patients experienced newly developed or aggravated musculoskeletal pain since taking AIs; (2) the relationships among the main variables; and (3) factors associated with AI continuation intention.

## Methods

Ethics statement: This study was approved by the Institutional Review Board of Korea National Cancer Center (No. NCC2019-0235). Informed consent was obtained from the participants.

### Design and participants

This cross-sectional correlational research was conducted at a cancer center located in Goyang, Gyeonggi Province, Korea, from September 16 to November 14, 2019. The participants were recruited via convenience sampling among patients visiting the outpatient clinic of the center for breast cancer. Based on the findings of a previous study that AI-associated musculoskeletal pain started after 6 weeks on average [16], the inclusion criteria were as follows: (1) stage I–III breast cancer; (2) completed surgery, adjuvant chemotherapy, and radiation therapy; and (3) at least 6 weeks on AI medication [16]. BCS who were treated for other cancers and metastatic breast cancer (stage IV) were excluded to rule out other causes of musculoskeletal pain. The sample size needed for multiple linear regression, calculated using G*Power 3.1.9.2 (α error probability=.05, statistical power=90%, effect size=.15 [17], and four predictors: pain severity [7,11], pain interference [11,18], FCR [4], and beliefs about endocrine therapy [4,12]), based on the literature was at least 108. Considering a 20% dropout rate, questionnaires were distributed to 129 BCS and 123 completed questionnaires without missing information were included in the analyses.

### Assessment tools

A self-reported questionnaire including AI continuation intention, musculoskeletal pain, FCR, beliefs about endocrine therapy, and general characteristics was used. Permission to use each assessment tool was obtained from both the developers and the authors of adapted/translated versions by e-mail.

#### Aromatase inhibitor continuation intention

A five-item measure of intentions to take cardiac medication [6] was modified to evaluate AI continuation intention, with permission from the developer. For example, the item “I plan to take regular medication in the future” was modified to “I plan to continue endocrine therapy in the future.” The five items evaluated intentional plan to take medication, intention to make an effort to take the medication, intention to persist and adhere with the medication, and the perceived possibility of taking the medication. Each item is rated on a 5-point scale (1–5), with a possible total score ranging from 5 to 25, and higher scores indicate greater intention. Cronbach’s α was .88 at the time of development [6] and .98 in this study.

#### Musculoskeletal pain

Musculoskeletal pain was evaluated with the Korean version [19] of the Brief Pain Inventory-Short Form (BPI-SF) [20]. Pain severity (four items) and pain interference (seven items) are rated on an 11-point scale (0–10), with a higher score indicating more severe pain and pain interference. Cronbach’s α was .80 to .92 at the time of development [20] and was .89 for pain severity and .94 for pain interference in this study.

#### Fear of cancer recurrence

FCR was evaluated with the Fear of Cancer Recurrence Inventory-Short Form (FCRI-SF) [21] Korean version [22], which consists of nine items rated on a 5-point scale (0–4), with a possible total score range of 0 to 36 and a higher score indicating a greater FCR. A cutoff score of 13 or higher is considered clinically significant [21]. Cronbach’s α was .89 at the time of development [23], .77 in a study with a Korean population [22], and .83 in this study.

#### Beliefs about endocrine therapy

The 22-item Endocrine Therapy Beliefs Scale [24], which was developed in Korean, was used to measure the cognitive response to endocrine therapy. It consists of four subcategories: perceived control (11 items), perceived concerns (four items), perceived benefits and trust (five items), and perceived logic (two items). Each item is rated on a 4-point scale (1–4), with a possible total score range of 22 to 88, and a higher score indicates a stronger beliefs about endocrine therapy. Cronbach’s α was .91 at the time of development [24] and .90 in this study.

#### General characteristics

A short questionnaire was developed to collect information about demographic and disease-related characteristics relevant for AI adherence [4,12]. General characteristics included age, cancer stage, breast cancer treatment, time since menopause, body mass index, and history of musculoskeletal disease.

### Data analyses

The data were analyzed using IBM SPSS ver. 25.0 (IBM Corp., Armonk, NY, USA). Differences in AI continuation intention according to musculoskeletal pain were examined using Welch analysis of variance. Correlations among variables were examined using Pearson correlation coefficients, and regression analysis was done to identify factors influencing AI continuation intention.

## Results

### General characteristics

The demographic and clinical characteristics of the 123 respondents are shown in Table 1. The mean age of the participants was 58.06±7.41 years, and they ranged in age from 30 to 80 years old. Among AI medications, letrozole (n=89, 72.4%) was prescribed twice as often as anastrozole (n=34, 27.6%), reflecting clinical practice. The mean duration of AI medication was 2.55±1.54 years and two participants had been taking AI for longer than 5 years. More than one-third of participants (n=44, 35.8%) had been diagnosed with musculoskeletal diseases and 27 of them (61.4%) reported that the diagnosis was made after initiating AI medication.

### Aromatase inhibitor continuation intention

The mean score of AI continuation intention was 22.28±4.34, which is interpreted as high (Table 2).

### Musculoskeletal pain, fear of cancer recurrence, and beliefs about endocrine therapy

In total, 107 participants (87.0%) reported that they experienced newly developed or aggravated musculoskeletal pain after the commencement of AI. The score for the worst pain within 24 hours was 5.08±2.80, indicating moderate pain requiring active pain management, and the mean score for the worst, the least, the average, and present pain was 3.29±2.12, indicating mild pain. Musculoskeletal pain was most commonly reported as interfering with mood (4.39±2.90), sleep (4.26±3.32), and general activities (4.11±3.28) (Table 2).

The mean score for FCR was 17.62±7.14, and 74.0% of participants had clinically significant FCR (≥13). The mean score for beliefs about endocrine therapy was 67.54±9.49, which is interpreted as high (Table 2).

### Aromatase inhibitor continuation intention according to musculoskeletal pain

To evaluate the difference in AI continuation intention according to pain severity, the mean scores for the worst, the least, the average, and present pain were classified into three groups; none (score of 0), mild (1-3), and moderate (4-7). There was no mean score greater than 7. The scores for the worst pain within 24 hours were classified into four groups—none (score of 0), mild (1–3), moderate (4–7), and severe (8–10) [25]—because AI continuation intention may be most strongly affected by the worst pain, and the use of a single item for the worst pain severity is also supported by the BPI user guide [26]. The analysis showed that there were no significant differences in AI continuation intention between the groups (Table 3).

### Relationships among aromatase inhibitor continuation intention, musculoskeletal pain, fear of cancer recurrence, and beliefs about endocrine therapy

The correlations among variables are shown in Table 4. A moderate correlation was found between AI continuation intention and beliefs about endocrine therapy (r=.66, *p*<.01). FCR had a weak relationship with both pain severity (r=.21, *p*<.05) and pain interference (r=.35, *p*<.01). Beliefs about endocrine therapy had a weak relationship with pain interference (r=–.18, *p*<.05)

### Beliefs about endocrine therapy as a factor influencing aromatase inhibitor continuation intention

Beliefs about endocrine therapy was the only factor that influenced AI continuation intention. The regression model was statistically significant (F=95.66, *p*<.001), explaining 44% of AI continuation intention (Table 5). The Durbin-Watson value was 2.027, implying independence of the residual without autocorrelation. The normality of residuals was examined using a histogram and a normal probability plot.

## Discussion

Beliefs about endocrine therapy was the only factor with a statistically significant effect on AI continuation intention in this study of BCS who reported newly developed or aggravated musculoskeletal pain since taking AI. This result is comparable to results of previous studies, conducted among BCS either taking AI or tamoxifen, according to which beliefs about endocrine therapy was the most powerful factor affecting medication adherence [4,12] and was associated with intention to take endocrine therapy [27]. It is also consistent with the finding of a qualitative study that breast cancer patients continuously reassess the necessity of endocrine therapy, its risks, and other options during endocrine therapy, and if they become doubtful about it efficacy they are likely to make a decision to stop taking the medication [3]. The findings of this study thus underscore the need to develop interventions for maintaining and reinforcing beliefs about endocrine therapy during the period which it is prescribed, which is usually 5 years. Further studies that can identify intervention time points, methods, and screening techniques for vulnerable populations will also be beneficial.

The majority of the participants (87%) experienced newly developed or aggravated musculoskeletal pain after taking AI, which is a higher proportion than found in a previous study (73.7%) [28], and the worst pain severity within 24 hours was 5.08±2.80, suggesting the need for active pain control management. Musculoskeletal pain severity showed a significant positive correlation with pain interference and FCR. Thus, a multidisciplinary approach is needed for pain management for newly developed or aggravated musculoskeletal pain after taking AI.

Nevertheless, no significant relationship was found between musculoskeletal pain and AI continuation intention, unlike previous studies conducted in other countries [7,8,18]. In a study done in the USA, musculoskeletal symptoms were highly associated with early discontinuation of AI (hazard ratio, 4.39; 95% CI, 2.4–8.02; *p*<.0001) [18] and in a study done in Australia, 68% of BCS reported AI discontinuation due to AI-related musculoskeletal pain [8]. In addition, a prior study done in the USA reported that a moderate or higher worst pain score (>4 on the BPI) was a predictor of premature discontinuation of AIs [7]. The reason for this discrepancy may be related to the high levels of AI continuation intention in the present study, the difficulty in making direct comparisons due to prior studies’ focus on continuation behaviors rather than intention, and the discrepancy between intention and behavior. In a previous study conducted to identify predictors of thoughts about stopping endocrine treatment (either tamoxifen or AIs), 30% of the participants reported that they considered discontinuing endocrine therapy in addition to the 36% of participants who had already stopped endocrine therapy, and symptom severity was most strongly associated with these thoughts [5]. A direct comparison is difficult because different measurement tools were used and 39% of participants in the previous study were premenopausal women, which may affect symptom severity. However, the intention to continue AIs among those who either discontinued or considered stopping endocrine therapy in that study [5] was presumably lower than this study’s level of 22.28±4.34. Although previous studies [7,18] of AI (non)adherence focused on behavior rather than intention, an accurate identification of BCS who are indecisive about AI continuation would be helpful for preventing premature discontinuation and encouraging BCS to complete the therapy during the recommended period. Thus, further research should develop valid tools evaluating intention to continue endocrine therapy and seek to identify vulnerable populations. In addition, the discrepancy between intention and behavior may be relevant. In this study, AI continuation intention was measured instead of adherence (i.e., a behavioral variable), considering the lack of reliability and evidence regarding self-reported measures of medication adherence [29]. Although intention to take medication is a predictor of medication adherence [6], actual behavior can differ. Thus, other factors should be considered when using intention as a predictor of behavior, and further studies with a longitudinal design are required to evaluate the predictive power of intention on adherence in the context of endocrine therapy with AI.

A significant issue is that 74.0% of participants reported clinically significant FCR (≥13) and the average FCR score (17.62±7.14) was also higher than the result of a previous study conducted among patients taking AIs (14.8) [14]. Despite the moderate pain severity, the finding of a high degree of FCR in this study is worthy of notice. FCR also had a weak relationship with pain severity and pain interference. This result implies that strategies to deal with FCR should be included in developing multidisciplinary nursing interventions for BCS. Although FCR in this study was higher than found in a previous study, it had no effect on AI continuation intention, which may be related to the high level of beliefs about endocrine therapy found in this study. Since it has been noted that FCR plays a role in endocrine therapy continuation among BCS with low levels of beliefs about endocrine therapy [4], further research should explore this interplay.

This cross-sectional study has several limitations. First, generalizability is limited as participants were recruited via convenience sampling from a single institution. Second, the discrepancy between AI continuation intention and AI adherence behavior was not evaluated. Although intentions explain behaviors to some extent, the predictability of actual AI continuation was not evaluated, as AI-taking behavior was not the focus of this study. Third, measurement issues may need to be considered, as the BPI-SF was not specifically developed to assess AI-related musculoskeletal pain and BCS often experience upper body discomfort and neuropathy after surgery, chemotherapy, and radiation therapy. While the researcher emphasized the aim of assessing newly developed or aggravated musculoskeletal pain after taking AI to improve the accuracy of the assessments, it is possible that the measurements may have only partially captured the actual pain that BCS experienced.

Despite the limitations, this study is meaningful from two perspectives. The findings present the current status of AI-related musculoskeletal pain, as self-reported by Korean BCS; to our knowledge, this is a novel contribution. Second, the evaluation of the relationship of musculoskeletal pain to AI continuation intention in a Korean population is also significant, since data indicating low AI adherence have been published from other countries, but the relevance of those findings to nursing practice in Korea has not been clear. This study’s finding can improve clinicians’ understanding of musculoskeletal pain during AI therapy and can be used to develop nursing interventions for musculoskeletal pain management and improving endocrine therapy continuation intention.

In conclusion, beliefs about endocrine therapy was a significant predictor of AI continuation intention, whereas musculoskeletal pain during AI therapy did not negatively affect AI continuation intention. The incidence of the pain and the pain severity, however, was high enough to require active pain management in this sample of BCS. Pain also had a moderate positive relationship with pain interference, and had a positive, albeit weak, relationship with FCR. Thus, there is a need to raise awareness and educate patients about the incidence and the severity of AI-related musculoskeletal pain and its impact, through educational programs or by distributing educational resources. Nursing interventions that support and reinforce beliefs about endocrine therapy are needed, and incorporating multidisciplinary pain management may be beneficial.

## Figures and Tables

**Table 1.** General characteristics of the participants (N=123)

Variable	Categories	Mean±SD (range)	n (%)
Age (year)		58.06±7.41 (30–80)	
	<50		13 (10.6)
			
	50–59		65 (52.8)
	≥60		45 (36.6)
Cancer stage	Stage I		55 (44.7)
	Stage II		41 (33.3)
	Stage III		27 (22.0)
Chemotherapy	Yes		68 (55.3)
	No		55 (44.7)
Radiation therapy	Yes		120 (97.6)
	No		3 (2.4)
Type of AI	Letrozole		89 (72.4)
	Anastrozole		34 (27.6)
Duration of medication (year)		2.55±1.54	
	<1		25 (20.3)
	1–2.99		47 (38.2)
	3–4.99		49 (39.8)
	≥5		2 (1.6)
Previous use of tamoxifen	No		107 (87.0)
	Yes		16 (13.0)
Time since menopause (year)		8.50±6.92	
	<5		42 (34.1)
	5–9		40 (32.5)
	≥10		41 (33.3)
Body mass index (kg/m^2^)		23.49±3.10	
	Normal (<23)		63 (51.2)
	Overweight (23–24.9)		22 (17.9)
	Obese (≥25)		38 (30.9)
MSD	No		79 (64.2)
	Yes		44 (35.8)
Diagnostic point of MSD	Before AI		17 (38.6)
	After AI		27 (61.4)

AI: Aromatase inhibitor; MSD: musculoskeletal disease.

**Table 2.** Aromatase inhibitor (AI) continuation intention, musculoskeletal pain, fear of cancer recurrence, and beliefs about endocrine therapy (N=123)

Variable	Possible score	Mean±SD		n (%)	Range
AI continuation intention	5–25	22.28±4.34	123 (100)		5–25
Musculoskeletal pain					
Pain severity^†^	0.25–10	3.29±2.12		107 (87.0)	0.50–7.00
Worst pain during the last 24 hours	1–10	5.08±2.80			1–10
Average pain	0–10	3.96±2.36			1–8
Least pain during the last 24 hours	0–10	2.17±1.91			0–6
Present pain	0–10	1.93±2.57			0–9
Pain interference^‡^	0.14–10	3.82±2.66		107 (87.0)	0.14–9.29
Mood	0–10	4.39±2.90			0–10
Sleep	0–10	4.26±3.32			0–10
General activities	0–10	4.11±3.28			0–10
Usual work	0–10	4.06±3.21			0–10
Enjoyment of life	0–10	3.81±3.29			0–10
Walking ability	0–10	3.38±3.15			0–10
Interpersonal relationships	0–10	2.75±3.24			0–10
Fear of cancer recurrence	0–36	17.62±7.14		123 (100)	4–35
Clinical level (≥13)	13–36			91 (74.0)	
Nonclinical level	0–12			32 (26.0)	
Beliefs about endocrine therapy	22–88	67.54±9.49			38–88

† Pain severity=(worst+least+average+present pain)/4;

‡ Pain interference=sum of seven-item scores/7.

AI: Aromatase inhibitor.

**Table 3.** Differences in aromatase inhibitor continuation intention according to pain severity (N=123)

Pain severity	Categories (score)	n (%)	Mean±SD	F	*p*
Total mean^†^	None (0)	19 (15.4)	21.95±5.46	0.37	.689
	Mild (1-3)	55 (44.7)	22.65±3.62		
	Moderate (4-7)	49 (39.8)	21.98±4.67		
Worst pain severity	Not at all (0)	17 (13.8)	22.41±5.32	1.04	.380
	Mild (1–3)	30 (24.4)	23.30±3.28		
	Moderate (4–7)	49 (39.8)	22.14±3.99		
	Severe (8–10)	27 (22.0)	21.30±4.34		

Number of severe pain category (score≥7) in total mean pain is zero.

† Total mean=(worst+least+average+present pain)/4.

**Table 4.** Correlations among study variables (N=123)

Variable	Pain severity	Pain interference	Fear of cancer recurrence	Beliefs about endocrine therapy
Pain severity	1			
Pain interference	.48**	1		
Fear of cancer recurrence	.21*	.35**	1	
Beliefs about endocrine therapy	–.05	–.18*	.09	1
AI continuation intention	–.03	–.04	.01	.66**

AI: Aromatase inhibitor.

* *p*<.05,

** *p*<.01.

**Table 5.** Factors influencing aromatase inhibitor continuation intention (N=123)

Variable	Model				
	B	SE	β	t	*p*
(constant)	1.73	2.12		.82	.42
Beliefs about endocrine therapy	.31	.03	.66	9.78	<.001
F (*p*)=95.66 (<.001), R^2^=.437, adjusted R^2^=.442					

B: unstandardized; β: standardized coefficient.
